# Student Perspectives on the Efficacy of Hybrid Simulation Laboratory Teaching‐Practicing Modules

**DOI:** 10.1111/eje.70044

**Published:** 2025-08-20

**Authors:** Jing Li, Xin Lin, Xiaoli Lian, Xiaodong Chen, Zhitao Wang, Chunxia Chen, Feifei Ma, Yao Chen, Yanmei Dai, Huiru Zou

**Affiliations:** ^1^ Tianjin Key Laboratory of Oral and Maxillofacial Function Reconstruction, Tianjin Stomatological Hospital The Affiliated Stomatological Hospital of Nankai University Tianjin China; ^2^ School of Medicine Nankai University Tianjin China; ^3^ Tianjin Medical College Tianjin China

**Keywords:** dental education, online teaching, remote guidance, teaching‐practicing module

## Abstract

**Introduction:**

This study examined dental students' perspectives on a hybrid simulation‐laboratory (sim‐lab) course for ultrasonic supragingival scaling.

**Materials and Methods:**

An interactive online system facilitated the course for second‐year dental students from 23 colleges. The curriculum encompassed demonstrations of ultrasonic scaling techniques, coupled with opportunities for students to pose inquiries and engage in remotely guided practice sessions. Upon course completion, an anonymous survey was administered, and the collected data were analysed using Pearson correlation coefficient analysis. Responses to the open‐ended question were tabulated and visualised through word clouds.

**Results:**

A total of 529 students participated, yielding 516 valid questionnaires. The majority of students (492) expressed a willingness to engage in online courses during their free time, with a mean score of 3.295 ± 0.577. Similarly, 511 students reported satisfaction with the course content selection, achieving a mean score of 3.490 ± 0.549. Furthermore, 505 students concurred that remote guidance was instrumental in refining their practical skills, scoring a mean score of 3.669 ± 0.529. Notably, only a minority of participants (0.78%, 0.78% and 0.39%, respectively) strongly disagreed with these assertions. A robust correlation was observed between satisfaction with the course content selection and improvements in practical skills (*r* = 0.541, *p* < 0.001). Conversely, students' willingness to study or explore online courses in their leisure time exhibited a weaker correlation with practical skill improvements (*r* = 0.269, *p* < 0.001).

**Conclusion:**

The findings of this study underscore the overwhelming acceptance of the hybrid sim‐lab course among dental students and its potential to elevate their educational journey.

## 1 Introduction

Periodontitis remains one of the most prevalent inflammatory diseases, with its severity and incidence steadily increasing over the past three decades [1]. As a fundamental periodontal treatment and dental hygiene maintenance method, ultrasonic supragingival scaling occupies a pivotal role in dental hygiene education [2, 3]. It constitutes an indispensable part of the clinical curriculum and forms a vital component of dental hygiene programmes. Consequently, it is imperative to offer a comprehensive ultrasonic supragingival scaling course to ensure students acquire the requisite skills [4].

Traditionally, preclinical simulation laboratory (Sim‐lab) training has involved direct, face‐to‐face interactions with faculty, providing students with hands‐on experience and personalised guidance [5]. However, in recent years, online education has gained widespread acceptance, offering lectures, case studies and problem‐based learning tutorials [6]. While some studies suggest that online education can be equally or even more effective than traditional methods [7, 8], many students still prefer the traditional face‐to‐face learning environment [9]. The primary challenges in online learning often revolve around the lack of essential facilities and limited interaction between teachers and students, highlighting the need for innovative approaches to engage and motivate students in online classes [10].

To address these challenges, a hybrid sim‐lab teaching‐practicing module for delivering the ultrasonic supragingival scaling course was developed and subsequently evaluated through feedback received from dental students. Utilising an anonymous questionnaire survey, we aimed to gain insights into students' perceptions of the effectiveness of online demonstrations, communication, and remote guidance. Furthermore, we collected feedback from open‐ended questions to obtain valuable insights, optimise online teaching methods, and ultimately enhance the learning outcomes for students. By examining these aspects, we aspire to contribute to the evolving landscape of online dental education and inform future endeavours in this field.

## 2 Materials and Methods

### 2.1 Study Design

A single‐arm prospective cohort study was conducted to assess the feasibility and effectiveness of a hybrid sim‐lab teaching‐practicing module for ultrasonic supragingival scaling. This study design did not involve a control group; the primary focus was on evaluating the implementation and student perspective of the online teaching approach.

### 2.2 Ethical Considerations

The study was approved by the ethics committee of the Union of Medical College (Approval No. PH2022‐B‐004) on 4 April 2022.

### 2.3 Participant Selection

All second‐year dental students from 23 Medical Colleges who possessed access to electronic equipment and a stable internet connection were invited to participate in this study. The selection criteria were designed to ensure that all participants had the necessary prerequisites to engage in the online learning process.

### 2.4 Teacher Training and Schedule Coordination

Prior to the commencement of the study, the main teacher from Tianjin Stomatological Hospital and 55 assistant teachers from the participating colleges underwent comprehensive training sessions. The objective of this training was to standardise the teaching approach and ensure consistency across all colleges. Additionally, the schedules of all colleges were coordinated to ensure that all students attended the same class simultaneously, regardless of their geographical location.

### 2.5 Establishment of the Hybrid Sim‐Lab Teaching‐Practicing Module

The hybrid sim‐lab teaching‐practicing module was structured into three distinct parts: lecture, demonstration and remote guidance.

- Lecture: A PowerPoint presentation was used to deliver the fundamental theoretical knowledge of ultrasonic supragingival scaling. The lecture was live‐streamed to students' mobile devices, iPads, or laptops. Students had the opportunity to pose questions either through real‐time comments or via the WeChat platform. The lecture duration was set at 15 min. Based on feedback received through the Bullet‐screen feature or comments collated by the assistant teachers, the main teacher could adjust the teaching content accordingly. The interactive recording and broadcasting system from SEEWO (Guangzhou Shirui Electronic Technology Co. Ltd., China) automatically recorded the lecture, enabling students to replay it anytime after the class.
- Demonstration: Following the lecture, the main teacher, assisted by a nurse teacher, demonstrated four‐handed ultrasonic supragingival scaling on a phantom head. This demonstration session lasted for 45 min. During the demonstration, both teachers continuously monitored the live streaming effects through multiple monitors placed around them, ensuring optimal visibility and demonstration quality. The SEEWO interactive recording and broadcasting system captured the demonstration with exceptional clarity, providing students with an in‐depth view of every aspect of the procedure. Various camera angles, including wide, medium shots, close‐ups and point‐of‐view shots, immersed students fully in the learning experience.

As the main teacher performed ultrasonic supragingival scaling on the phantom head during the live streaming demonstration, she provided detailed commentary on each step of the procedure. The wide and medium shots effectively showcased the comprehensive patient reception process, encompassing consultation, examination, diagnosis, treatment planning and pretreatment communication. The demonstration also clearly illustrated the correct positioning required for ultrasonic supragingival scaling.

The nurse teacher complemented the demonstration by introducing the instruments used for ultrasonic supragingival scaling and emphasising the importance of four‐handed operation. The close‐ups and point‐of‐view shots allowed students to observe the intricacies of the procedure, enhancing their understanding.

The main teacher then demonstrated the proper technique for holding the ultrasonic supragingival scaling instrument, explaining the finger rest requirements and demonstrating how to locate the working end of the instrument. Key points were emphasised, including the adjustable power settings of the ultrasonic scaler and their impact on the final therapeutic outcome. She also cautioned students about the displacement amplitude of the scaling tip, emphasising its potential to affect scaling efficiency and even cause damage to the root surface.

Through demonstration, the main teacher effectively utilised an ultrasonic scaler to remove artificial calculus from a typodont in a controlled, simulated clinical setting. This allowed her to demonstrate the crucial requirements of contact between the working end of ultrasonic tips and the tooth surface, as well as the instrument's moving direction and sequence. The students were able to gain a comprehensive understanding of the procedure, enhancing their skills and knowledge in ultrasonic supragingival scaling.

C Remote Guidance: After the demonstration, students had the opportunity to practice ultrasonic supragingival scaling on phantom heads under the guidance of their respective assistant teachers and remote guidance from the main teacher. Utilising a real‐time connection, the main teacher could closely observe the students' operations and offer remote guidance accordingly. She promptly pointed out any issues at each step and instructed the students on the correct usage of the instruments.

Within a span of 30 min, the main teacher engaged in face‐to‐face communication with the students via the cloud. The monitor camera's angle and range could be adjusted at any moment, ensuring that the students' posture and practice were clearly presented to both the main teacher and all the assistant teachers.

**FIGURE 1:**
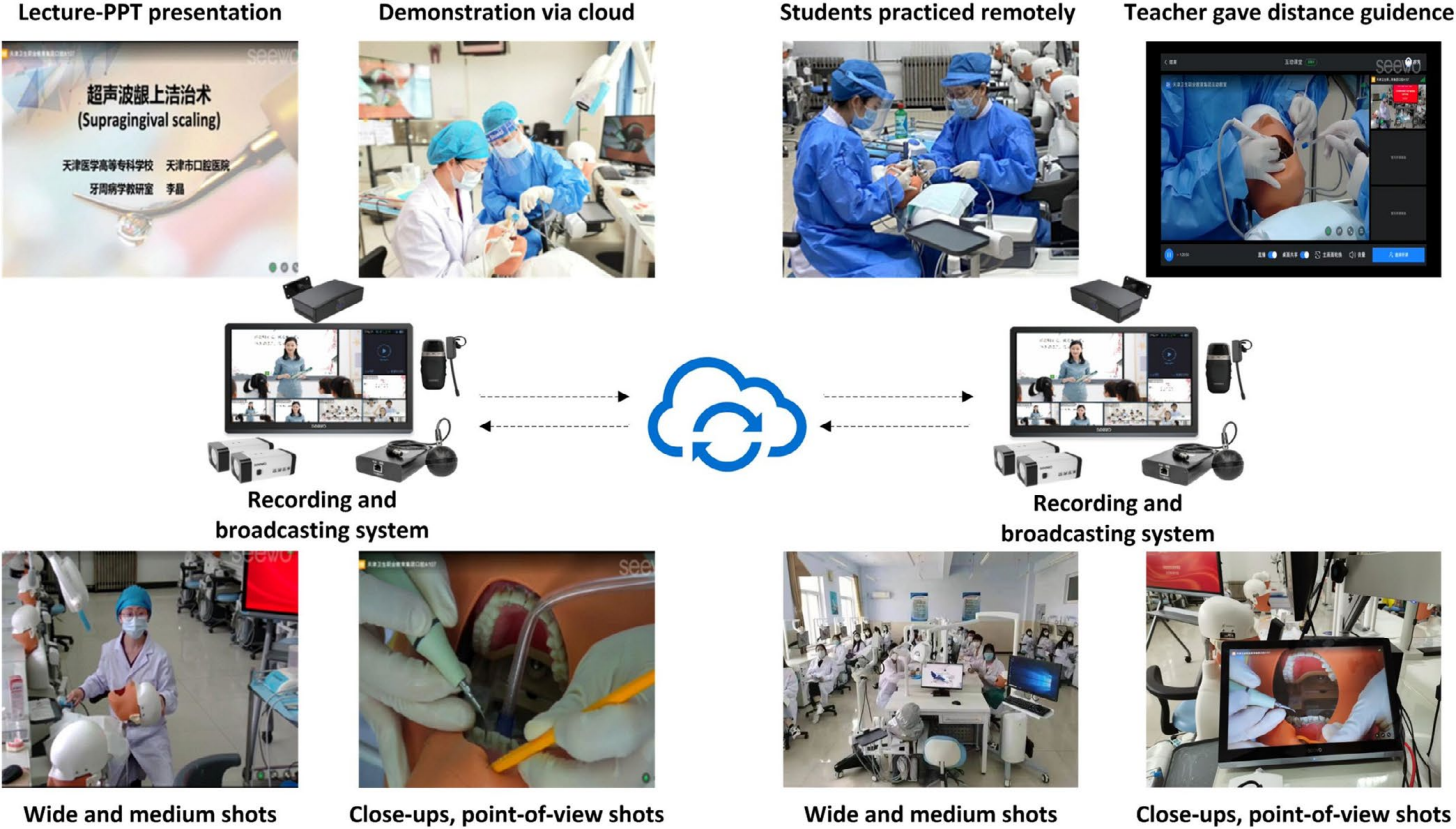
The hybrid sim‐lab ultrasonic supragingival scaling course. The teacher delivered lectures and demonstrations with the assistance of the recording and broadcasting system. The presentation included wide shots, medium shots, close‐ups and point‐of‐view shots as appropriate. Following this, students engaged in remote practice under the same recording and broadcasting system. The teacher provided guidance to students from a distance, and mutual interaction occurred without any barriers.

Throughout the entire process, students were encouraged to ask questions and interact with the teachers at any point. Additionally, the system allowed students to revisit and re‐check their practice repeatedly after class, aiding them in self‐discovery of potential issues and deepening their comprehension of the techniques. This approach ensured an effective and impactful learning experience for the students (Figure 1).

### 2.6 Data Collection and Analysis

Immediately after the class, an anonymous questionnaire survey with 16 four‐point Likert scale questions was conducted to access students' perspectives on online demonstration, communication, and remote guidance effectiveness. Teachers unrelated to the course design handled and collected questionnaires. Questionnaires with any unanswered questions were deemed invalid. Data was entered into Microsoft Excel 365 using a double entry method. Descriptive statistics summarised satisfaction, and a Pearson correlation coefficient analysis using SPSS software was conducted to explore the relationship between students' attitudes and practical skill improvement. An open‐ended question at the end gathered students' opinions and suggestions for course improvement.

## 3 Results

### 3.1 Participant Composition

**FIGURE 2:**
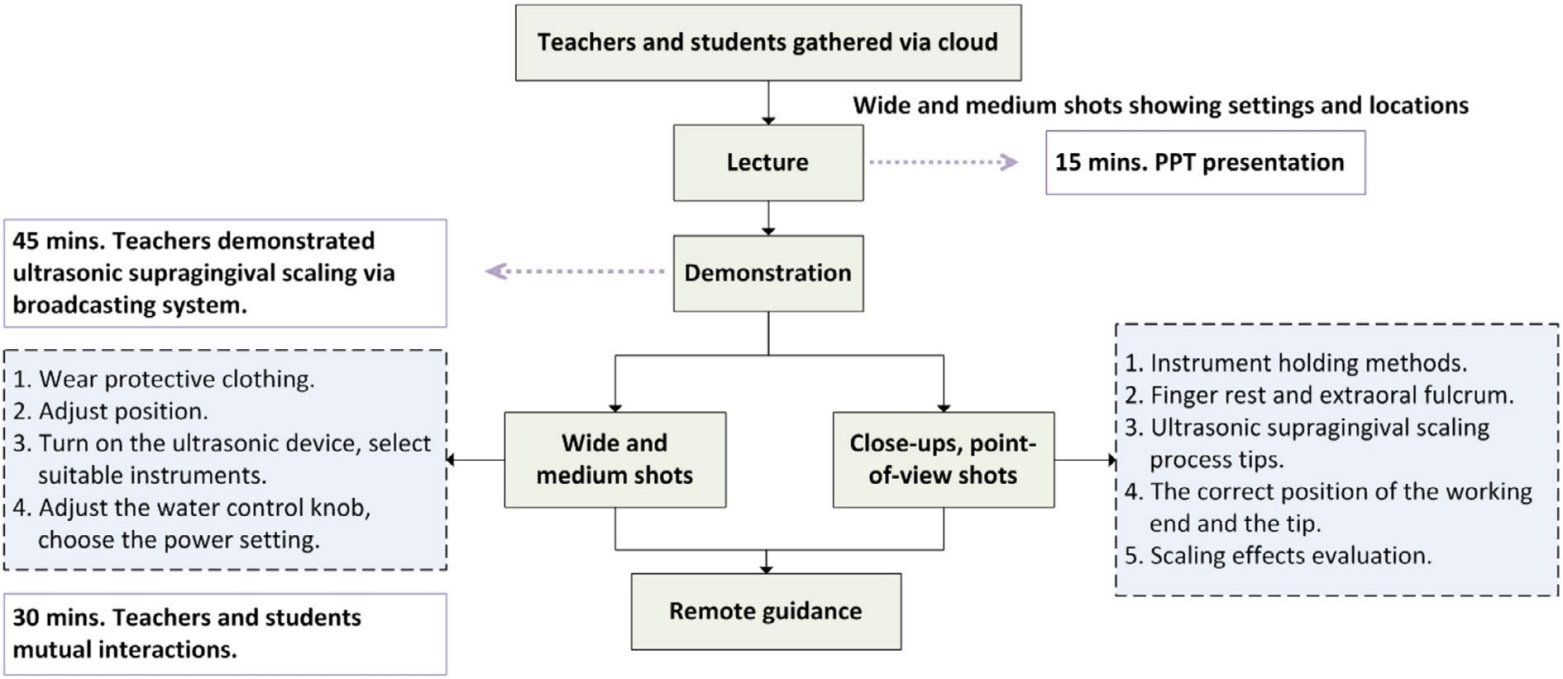
Flowchart of the online interactive teaching module for ultrasonic supragingival scaling training. This figure presents a visual representation of the flowchart for the online interactive teaching module specifically developed for ultrasonic supragingival scaling training. It outlines the sequential steps involved in the teaching and practicing process, starting from the initial introduction to the module, progressing through various interactive components such as video demonstrations, practice sessions, and feedback loops, and culminating in guidance and final evaluation.

A total of 529 students from 23 medical colleges participated in the live online course, with 56 hailing from rural backgrounds. The live broadcast proceeded seamlessly without any interruptions, ensuring an uninterrupted learning experience for all attendees. The main teacher conducted the remote synchronous class in a live, interactive format, which was well received by the students (Figure 2).

The students overwhelmingly commended the clarity and fluidity of the live streaming broadcasting. They agreed that the transitions between different shots were executed smoothly and precisely, significantly enhancing their overall viewing experience. Furthermore, the lecture effectively emphasised the crucial and challenging aspects of the course, leaving a lasting impression on the students regarding the clinical diagnosis and treatment process of periodontitis.

### 3.2 Student Evaluation Survey Results

A total of 516 valid questionnaires were collected in this study, providing valuable insights into the students' perspectives. Among the participants, 76.16% were female, while 23.84% were male. The survey revealed that students had utilised various functions and services of the online system, with 77.71% engaging with live broadcasting, 74.61% utilising the replay feature, 65.12% participating in in‐class practising and 58.72% completing online testing (Table 1).

**TABLE 1:** Gender and online system experience survey.

Item	% (*N*)
Gender	
Male	24.00% (127)
Female	76.00% (402)
The function and services of the online system experienced	
Live Streaming of Courses	78.00% (411)
Course Replay	74.20% (391)
Classroom Exercises	65.30% (344)
Online testing	58.10% (308)
Others	17.80% (94)

Using a four‐point Likert scale, the survey findings indicated that students rated their ‘Willingness to study or browse online courses in spare time’ at 3.295 ± 0.577, their ‘Satisfaction with the selection of course content’ at 3.490 ± 0.549, and their perception of the ‘Usefulness of the remote guidance in improving practical skills’ at 3.669 ± 0.529 (Table 2). Notably, 492 students expressed their willingness to engage in online courses during their spare time, while 511 students were satisfied with the chosen course material. Furthermore, a significant majority of students agreed that the remote guidance was beneficial in improving their practical skills, with 69.33% strongly agreeing and 28.49% agreeing.

**TABLE 2:** Students' attitudes towards online sim‐lab teaching–practicing module.

Item	Mean	Std	*N*	SD	D	A	SA
				% (*N*)	% (*N*)	% (*N*)	% (*N*)
Willingness to study or browse online courses in your free time	3.295	0.577	516	0.78% (4)	3.88% (20)	60.47% (312)	34.88% (180)
Satisfaction with the selection of course content	3.490	0.549	516	0.78% (4)	0.19% (1)	48.64% (249)	50.78% (262)
Usefulness of the remote guidance in improving students' practical skills	3.669	0.529	516	0.39% (2)	1.74% (9)	28.49% (147)	69.33% (358)

*Note:* The questionnaire used a four‐point Likert scale, ranging from 1 (strongly disagree) to 4 (strongly agree).

Abbreviations: A, Agree; D, disagree; SA, strongly agree; SD, strongly disagree.

Our analysis revealed an intriguing correlation: students' satisfaction with the course content was strongly associated with improvements in their practical skills (*r* = 0.541, *p* < 0.001). However, their willingness to study or browse online courses in spare time showed only a weak correlation with practical skill improvements (*r* = 0.269, *p* < 0.001) (Table 3).

**TABLE 3:** Correlation between students' attitudes and their practical skill improvement in this course.

Item	1	2	3
1. Willingness to study or browse online courses in your free time	—		
2. Satisfaction with the selection of course content	0.434***	—	
3. Usefulness of the remote guidance in improving students' practical skills	0.269***	0.541***	—

***p < 0.001.

The open‐ended question elicited numerous valuable suggestions from students. Notably, 64.53% of them expressed a desire for future courses to be designed and delivered similarly to this study. They envisioned the main teacher becoming a prominent social media influencer, efficiently disseminating knowledge and skills through live streaming broadcasts. This would allow students to watch the teacher's demonstrations anytime and facilitate more convenient communication with teachers. Additionally, students advocated for the development of simpler equipment to enhance the usability of the online teaching–practicing module. They recognised the creativity and potential of fully utilising the network platform for online teaching. Fully leveraging the network platform for online teaching represents an innovative approach, particularly benefiting rural areas. Witnessing teachers from diverse colleges collaborating to uphold teaching quality was an enriching experience. This collaboration not only improved teachers' online teaching skills and proficiency but also sparked students' enthusiasm for learning and practicing. Furthermore, 64.53% of students recommended incorporating additional resources such as videos or animations to enrich the learning experience. The theoretical modules could seamlessly integrate platforms like MOOC, SPOC and other online resources such as XuetangX. Modern information tools, including Rain Classroom, WeChat, QQ and DingTalk, enable the delivery of online courses through live broadcasts and video recordings. It is crucial to formulate and refine the standards for the online teaching–racticing module, encompassing aspects such as class preparation, teaching, counselling, interaction, supervision and assessment.

**FIGURE 3:**
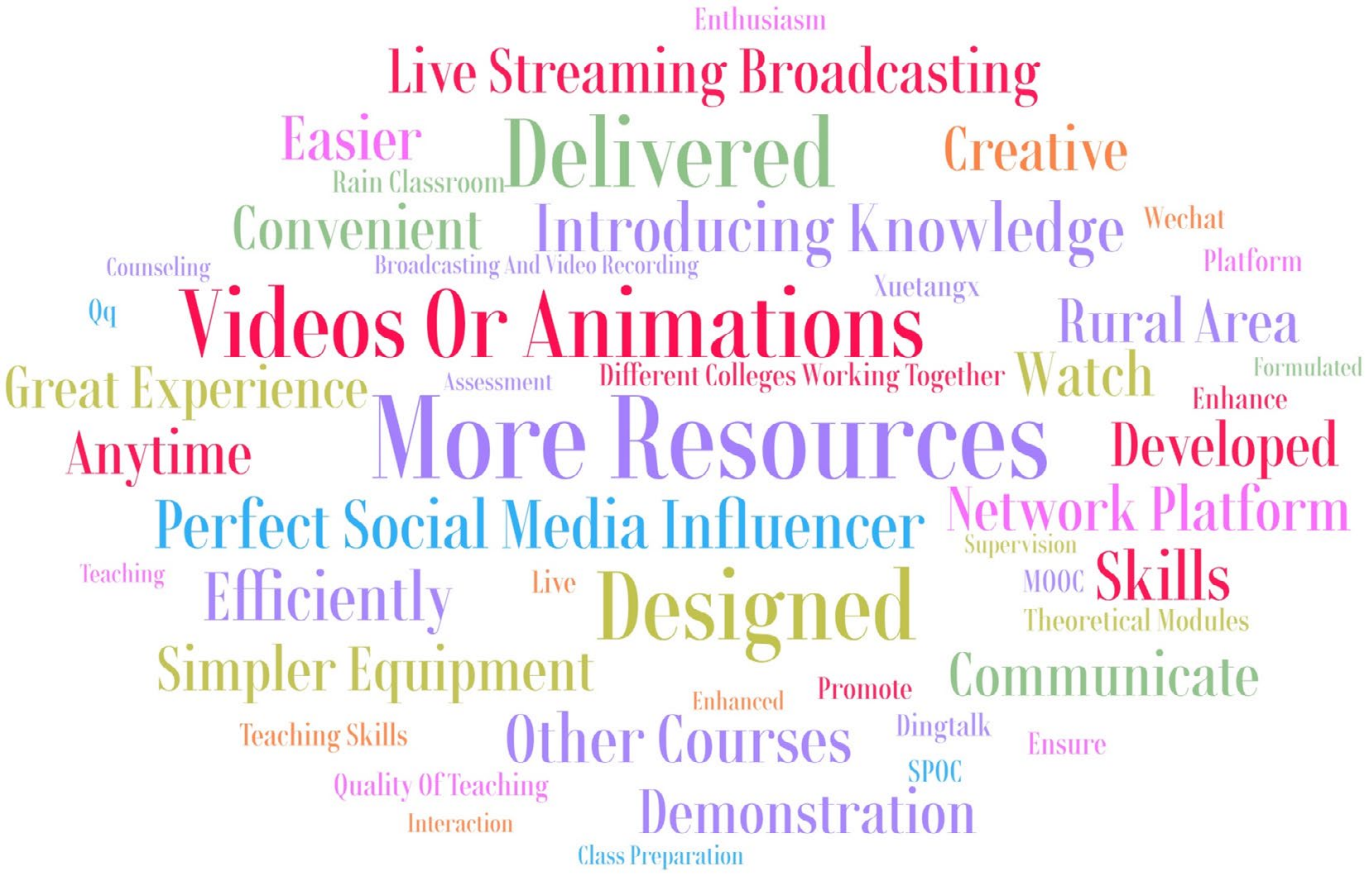
A word cloud visualisation generated from student responses to the open‐ended question. ‘More resources’ and ‘Videos or animations’ were the most frequently mentioned terms or phrases in the students' answers, with the size of these words indicating their frequency of occurrence.

Figure 3 displayed a word cloud visualisation generated from the responses provided by students to the open‐ended question. ‘More resources’ and ‘Videos or animations’ were the most frequently mentioned terms or phrases in the students' answers, with the size of these words indicating their frequency of occurrence.

## 4 Discussion

The conventional ultrasonic supragingival scaling practical training course typically comprises theoretical lectures, teacher demonstrations, hands‐on student practice, and teacher guidance. While this format fosters face‐to‐face instruction, easy communication, and minimal distraction, it presents inherent challenges [10]. Teachers often struggle to comprehensively elucidate the intricate details due to limited intra‐oral visibility, large class sizes and restricted time. Additionally, there are disparities in educational resources availability and teacher competencies across institutions. Some students, particularly those who are introverted or self‐conscious, may hesitate to engage or seek help in front of others [11], leading to reduced participation.

Fortunately, online teaching offers a solution to these obstacles. With the rapid advancement of information technology, the integration of technology into education has emerged as a pivotal focus in the modern era. Online education, leveraging digital techniques and network platforms [12], transcends the barriers of time and space, empowering learners to flexibly arrange their studies according to their preferences and convenience [10]. It is also economically efficient, broadening access to learning opportunities for a diverse range of students and potentially bridging the educational gap. This ensures that students in rural, impoverished, and remote areas can benefit from the same learning opportunities and access to the same educational resources as those in developed regions, promoting educational equity [13].

Online teaching has gained significant popularity in dental education, particularly during the COVID‐19 pandemic [14]. Our survey revealed that nearly all students have online learning experience and basic computer and internet skills. Al‐Natour et al. also reported that internet connection was not a barrier to online learning for most students (61.4%) [15]. Well‐designed online courses are available through platforms such as Tencent Meeting and Rain Class [16, 17]. These courses have garnered considerable attention from dental educators for enhancing teacher‐student connections and student focus.

Online education has revolutionised the teaching paradigm, shifting from a teacher‐centric to a more interactive, student‐centered approach. This transformation offers students the chance to gradually conquer their innate shyness and presents them with ample opportunities to articulate their thoughts and emotions, acquire fresh skills, and pose numerous inquiries. Research confirms that synchronous distance education is comparably effective to traditional education [18]. According to our questionnaire survey, students prefer diverse online learning activities, such as live broadcasting, replay functionality, in‐class practice and online assessments. Notably, live broadcasting was the most favoured option (Table 1). Overall, students have responded positively to online education, similar to previous reports [19, 20]. Several studies have also shown that [21, 22], online education offers unique advantages in enhancing students' knowledge and skills, making it a potential method for undergraduate medical teaching. Both students and teachers appreciate online education, especially for student‐teacher collaboration, signifying its potential for sustained incorporation into future curricula [9].

Practical training courses in dental education come with stringent requirements, necessitating teachers who possess not only solid theoretical knowledge and practical skills, but also possess strategies to ensure students fully grasp all details during training. In our study, remotely controlled cameras with adjustable settings were used, allowing teachers to select appropriate perspectives based on demonstration needs; thereby ensuring students obtained a comprehensive understanding. The results were impressive, with 95.35% of students eagerly anticipating participation in this online course. Another study explored a novel approach using active videography for online anatomy teaching [23]. The findings were noteworthy, as test scores for online practical teaching significantly surpassed those from traditional face‐to‐face teaching methods [23]. Additionally, the utilisation of simple, portable, and economical manikins allowed students to practice at any time and anywhere, attaching conveniently to desks. With the widespread availability of high‐tech devices and high‐speed internet, teachers can now provide both theoretical and methodological training guidance remotely. This accessibility is beneficial in the development of students' multi‐competencies.

Ultrasonic supragingival scaling is a fundamental technique that dental students must master [24]. Our questionnaire survey revealed that 99.42% of students expressed high satisfaction with their participation in this experimental course. Such positive feedback is encouraging and prompts us to conduct further investigations. Notably, at the end of the course, students imparted numerous insightful suggestions. Specifically, 64.53% of students proposed the integration of additional resources, such as videos or animations, to enrich the learning experience. However, despite the abundance of online video resources, many are disorganised and challenging for students to assess for reliability [25]. In this regard, the online resources meticulously curated and provided by professional teachers in schools gained the utmost trust from students [26].

Moreover, 43.99% of students desired more communication in online courses, suggesting offline messaging and live Q&A sessions after class. Various communication methods could be developed to enhance students' participation enthusiasm in future courses. Stimulating the enthusiasm of students is crucial in the learning process, especially in online education, which lacks face‐to‐face communication. A previous study showed that 77.7% of participants held a negative view of E‐learning [27]. This negative perception could stem from various factors, including absent in‐person interaction, communication challenges, poor internet connectivity increasing distractions and difficulty in sustaining focus. A study was conducted to investigate technology‐based learning (TB‐learning) among dental students during the pandemic outbreak of Corona Virus Disease 2019 [27]. Results revealed that the overall satisfaction and positive attitude towards TB‐learning among the students was less than 50%. One of the primary obstacles hindering the TB‐learning process is the lack of technical skills. A separate study examining dental students' responses indicated that their perception of TB‐learning was closely tied to their possession of essential computer skills and consistent internet access. Therefore, enhancing students' technical skills, understanding their online learning habits and preferred communication methods, and then adapting our teaching strategies accordingly, will greatly benefit students in the teaching‐practising module. This is particularly advantageous for students from rural areas, who can now access quality education through online platforms.

To ensure the success and uphold the high standards of online education, teachers must possess excellent communication, organisational and technological skills [27]. They must be passionate about teaching, proactive in guiding students, and responsive, dedicated, and timely in their interactions. By fully leveraging the unique characteristics of online education, strong bonds between teachers and students can gradually be forged. The attitudes and behaviours of teachers are very important when delivering online education to students. A warm and thoughtful approach in instruction and guidance is associated with an increase in students' feelings of preparedness and confidence [28]. Overall, it is worthwhile to explore the feasibility of online training in the future.

However, this study is inherently constrained by its non‐experimental design. Ethically, it was not feasible to randomly assign students from the same academic year to different groups and implement diverse educational models within the same class. Such a practice would be unjust, particularly since online education has proven to be beneficial. Another limitation is that our hybrid sim‐lab teaching–practicing module was only tested in a single course. Nevertheless, exploring its application in other courses could yield valuable insights, and additional comprehensive data may reveal further potential for integration in various scenarios.

Regarding the online course conducted using the interactive recording and broadcasting system, there are still a few notable limitations: Firstly, the complexity of the remote system necessitated ongoing technical support from personnel. This could potentially disrupt the flow of the course and require additional resources to ensure smooth operation. Secondly, coordinating with 23 medical colleges to align their schedules for a joint online session posed significant challenges. Achieving synchronisation across these institutions required extensive planning and coordination, which may not always be feasible or practical. Lastly, the online course placed high demands on network connectivity. Given the real‐time nature of the interactions and demonstrations, any disruptions in the network could compromise the quality of the course and the learning experience for the students [29, 30]. To mitigate these limitations, future implementations of similar online courses could explore simpler, more user‐friendly systems, foster greater institutional collaboration to facilitate scheduling, and invest in robust network infrastructure to ensure seamless connectivity.

## 5 Conclusion

The findings of this study suggested that, despite its inherent limitations, the hybrid sim‐lab teaching–practicing module could serve as an appropriate and accessible supplemental method for teaching practical dental skills. It presents a viable alternative to traditional classroom‐based dental education, offering a new avenue for enhancing student learning outcomes.

## Conflicts of Interest

The authors declare no conflicts of interest.

## Supporting information


**Data S1:** eje70044‐sup‐0001‐Supinfo1@Questionaire survey.docx.

## Data Availability

The data used in this study are publicly available and stored in https://force11.org/wp‐admin/edit.php?post_type=post.
