# Supplementary material for: Student Perspectives on the Efficacy of Hybrid Simulation Laboratory Teaching‐Practicing Modules
Source: Eur J Dent Educ. 2025 Aug 20;30(3):945–52. doi: 10.1111/eje.70044 (PMC13383370; doi:10.1111/eje.70044)
Supplement: Supplementary file 1 — Data S1: eje70044‐sup‐0001‐Supinfo1@Questionaire survey.docx. [file EJE-30-945-s001.docx]

**Tianjin Stomatological Hospital and Tianjin Medical College**

**Health Vocational Education "Double High" Construction Alliance Cloud Classroom Online Survey**

**(For Students)**

1. Your Gender: □ Male □ Female
2. Proficiency in Computer Operation:

□ Not Proficient □ Average □ Fairly Proficient □ Very Proficient

1. Have you ever received online learning? □ Yes □ No
2. Are you willing to actively study or browse online courses in your free time?

□ Very Willing □ Fairly Willing □ Not Willing □ Very Unwilling

1. Which type of course resources would you prefer to learn? (Multiple Choices Allowed)

□ Basic Theory □ Professional Theory □ Practical Training □ Clinical Practice

1. Which functions of online learning platforms do you frequently use? (Multiple Choices Allowed)

□ Live Streaming of Courses □ Course Replay □ Classroom Exercises □ Online testing □ Others

1. During the learning process, which method do you prefer to use for communicating with teachers and other classmates?

□ Text/Chat □ Voice □ Video □ Other

1. Compared with traditional courses, online course scheduling can be more flexible.

□ Strongly Agree □ Somewhat Agree □ Disagree Somewhat □ Strongly Disagree

1. How satisfied are you with the content selection of this course?

□ Very Satisfied □ Fairly Satisfied □ Dissatisfied □ Very Dissatisfied

1. How satisfied are you with the difficulty level of this course?

□ Very Satisfied □ Fairly Satisfied □ Dissatisfied □ Very Dissatisfied

1. How satisfied are you with the integrated design of theory and practical training in this course?

□ Very Satisfied □ Fairly Satisfied □ Dissatisfied □ Very Dissatisfied

1. Do you think the explanation and demonstration of the four-hand operation technique in this course will be helpful for your future studies and work?

□ Very Helpful □ Somewhat Helpful □ Not Helpful □ Not at All Helpful

1. Do you think the design of remote guidance for student operations in this course is helpful?

□ Very Helpful □ Somewhat Helpful □ Not Helpful □ Not at All Helpful

1. Do you think this course will be helpful for your future studies and work?

□ Very Helpful □ Somewhat Helpful □ Not Helpful □ Not at All Helpful

1. How satisfied are you with the overall arrangement of this course?

□ Very Satisfied □ Fairly Satisfied □ Dissatisfied □ Very Dissatisfied

1. What do you think are the shortcomings of this course? (Multiple Choices Allowed)

□ Course resources are repeated, similar to other courses

□ Course content design is unreasonable, not explained in depth

□ Time arrangement of various sections is unreasonable, too compact or too loose

□ After learning, there is no significant improvement in relevant knowledge and operational skills

□ Other (Please Specify)

1. What aspects do you hope will be improved in the course in the future? [Multiple Choices]

□ Continuous updating of course resources

□ Providing local downloads of instructional videos

□ Increasing more videos, animations, and other resources

□ Providing various types of reference materials that can be integrated into one's own teaching

□ Supporting different communication methods

□ Reducing resource access restrictions, preferably fully open access

□ Improving resource access speed

□ Other

1. What are your opinions and suggestions for this course training?
